# Reweighted multi-view clustering with tissue-like P system

**DOI:** 10.1371/journal.pone.0269878

**Published:** 2023-02-10

**Authors:** Huijian Chen, Xiyu Liu

**Affiliations:** Business School, Shandong Normal University, Jinan, China; University of Bradford, UNITED KINGDOM

## Abstract

Multi-view clustering has received substantial research because of its ability to discover heterogeneous information in the data. The weight distribution of each view of data has always been difficult problem in multi-view clustering. In order to solve this problem and improve computational efficiency at the same time, in this paper, Reweighted multi-view clustering with tissue-like P system (RMVCP) algorithm is proposed. RMVCP performs a two-step operation on data. Firstly, each similarity matrix is constructed by self-representation method, and each view is fused to obtain a unified similarity matrix and the updated similarity matrix of each view. Subsequently, the updated similarity matrix of each view obtained in the first step is taken as the input, and then the view fusion operation is carried out to obtain the final similarity matrix. At the same time, Constrained Laplacian Rank (CLR) is applied to the final matrix, so that the clustering result is directly obtained without additional clustering steps. In addition, in order to improve the computational efficiency of the RMVCP algorithm, the algorithm is embedded in the framework of the tissue-like P system, and the computational efficiency can be improved through the computational parallelism of the tissue-like P system. Finally, experiments verify that the effectiveness of the RMVCP algorithm is better than existing state-of-the-art algorithms.

## 1. Introduction

Membrane computing [1–5], as a branch of natural computing, aims to abstract computational models from the structure and function of biological cells and from the collaboration of cell groups such as organs and tissues. Membrane computing has been developed so far, and it mainly includes three basic computing models: cell-like P system [6], tissue-like P system [7, 8] and neuro-like P system [9, 10]. In the process of calculation, each cell acts as an independent unit, and each unit runs independently without interfering with each other [11]. The entire membrane system runs in extremely parallel mode. The tissue-like P system consists of cells and environment containing objects and rules. The movement of objects from cell to cell or cell to environment is carried out through the rules in extremely parallel execution. The tissue-like P system can be combined with other algorithms to improve the computational efficiency of the algorithm thanks to the computational parallelism of the tissue-like P system.

Clustering [12–15] is a tool of machine learning and artificial intelligence, which divides a group of data points into corresponding clusters, so that the similarity of data points in clusters is high, and lower similarity between clusters. It is an unsupervised learning technique. a great deal of single-view clustering methods have been proposed, such as spectral clustering [16–18], graph clustering [19, 20], subspace clustering [21], k-means clustering [22] and so on. With the deep research of clustering, the combination of clustering and deep learning methods and the application of clustering have been widely studied and achieved good clustering performance. Network clustering is related to many real applications, such as social community detection [23]; and disease module identification [24]. Wang et al. [25] proposed a single-cell clustering model based on denoising autoencoder and graph convolution network.

With the development of science and technology, more and more data are represented by multiple views, which are known as multi-view data. [26]. Compared with single-view clustering, multi-view clustering [27–32] has received extensive attention due to its better clustering performance. So far, a variety of multi-view clustering methods have been proposed. Multi-view clustering methods can be roughly divided into the following categories: multi-view k-means clustering [33], multi-view spectral clustering [34], multi-view subspace clustering [28, 30, 35], multi-view graph clustering [36, 37], multi-task multi-view clustering [38], etc. Multi-view subspace clustering and multi-view graph clustering have been widely studied owing to their satisfactory clustering performance. Self-representation model has achieved commendable progress in the study of single-view subspace clustering, which regards each data point as a linear combination of data. The subspace representation matrix **S**, which is also regarded as the similarity matrix, can be obtained as follows:

$$\begin{array}{c} \min_{S}∥X-XS{∥}_{F}^{2}+\alpha ∥S{∥}_{F}^{2} \end{array}$$ (1)

where **X** is the original data matrix. Guo et al. [39] extended the single-view self-representation model to multi-view clustering, which assumes that samples from different categories are embedded in independent subspaces. Therefore, the fused multi-view self-representation feature should be a block diagonal. The noise information in the data has always been the main factor affecting the clustering performance. In order to alleviate the impact of noise information on the clustering performance and make better use of the information of each view, scholars have proposed many methods. For example, Yin et al. [40] used a more direct and intuitive block diagonal regularization to preserve the underlying structure of each view, and at the same time introduced the Cauchy loss function to deal with noise information. The underlying public structure of multi-view data can be effectively retained by the derived consistency representation matrix, and is robust to noise information and data damage. In addition, the clustering performance will also be affected by the process of fusing the similarity matrix. Kang et al. [41] proposed a new multi-view clustering model in which the fusion graph approximates the original graph of each individual view but maintains an explicit cluster structure. The existing multi-view subspace clustering method still has a problem. After getting the similarity matrix of each view and the final uniform matrix, the second operation is implemented, that is, applying additional clustering algorithms (usually spectral clustering algorithms) to the uniform matrix, which will affect the clustering performance. Zhang et al. [42] proposed a Consensus One-step Multi-view Subspace Clustering model, which can solve the defect of poor clustering performance caused by the two-step operation.

Graph-based multi-view clustering method is one of the most popular multi-view clustering methods. In this method, the similarity matrix of each view is first constructed and merged into a unified matrix, and then an additional clustering algorithm or other methods are applied to the unified matrix to acquire clustering results. The construction of the similarity matrix of each view is a very significant step, the reason is that the quality of the similarity matrix of each view has a great impact on the final clustering performance. Many scholars have proposed some methods for constructing similarity matrix, such as k-nearest neighbor algorithm(k-NN), Clustering with Adaptive Neighbors [12], etc. The construction of similarity matrix is affected by many factors, such as noise information and outliers, similarity metrics, etc. Huang et al. [43] proposed a new model that simultaneously performs multi-view clustering tasks and learns similar relationships in the kernel space. If there are c clusters, the target optimal graph can be directly divided into precise c connected components. In addition, the model can automatically assign appropriate weights to each view without additional parameters. The allocation of weights is an important topic in machine learning. For example, Liu et al. [44] proposed a new weight initialization method. Weight allocation in multi-view clustering is also significant, and the method in this paper will focus on the weight allocation of each view.

**Fig 1:**
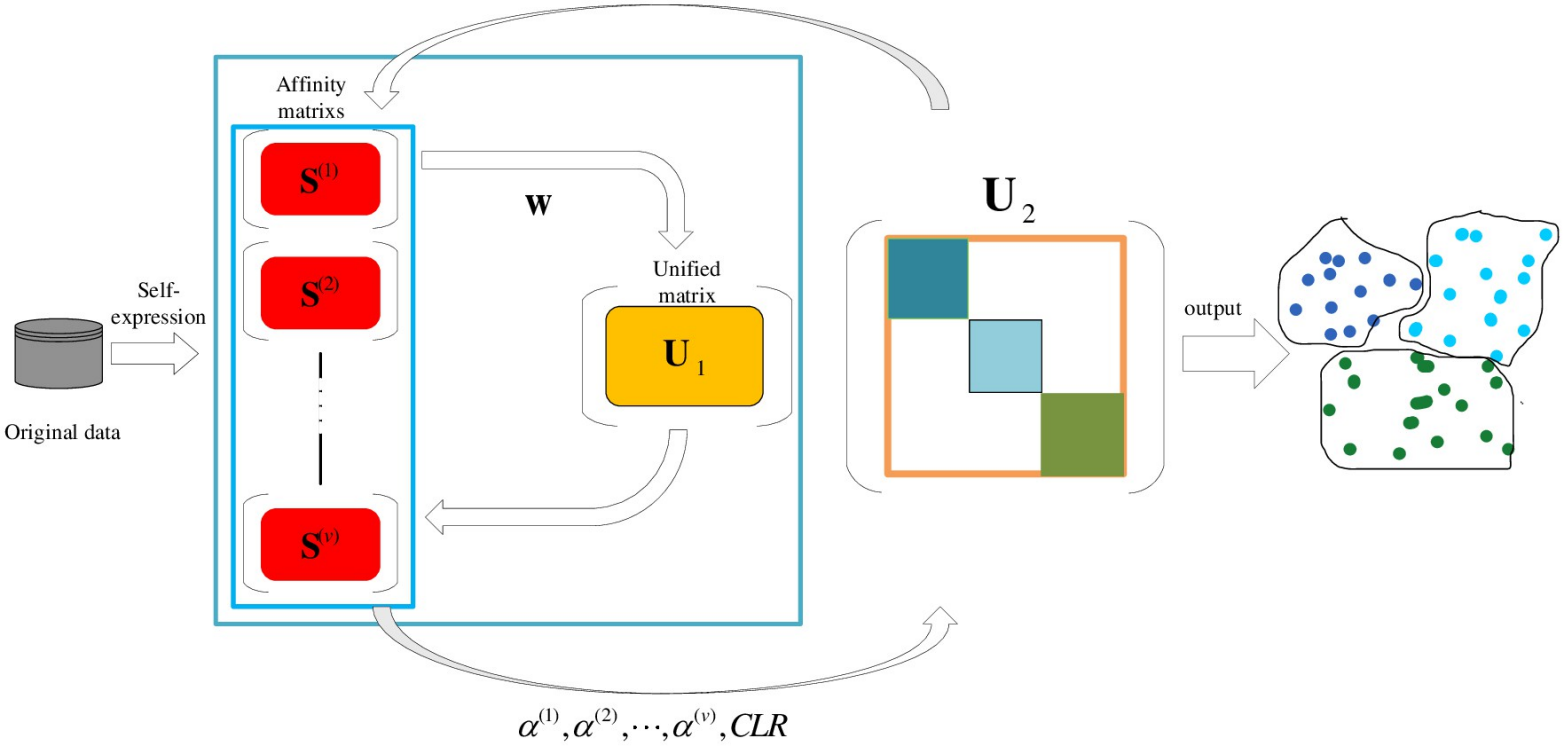
The RMVCP process without tissue-like P system.

**Fig 2:**
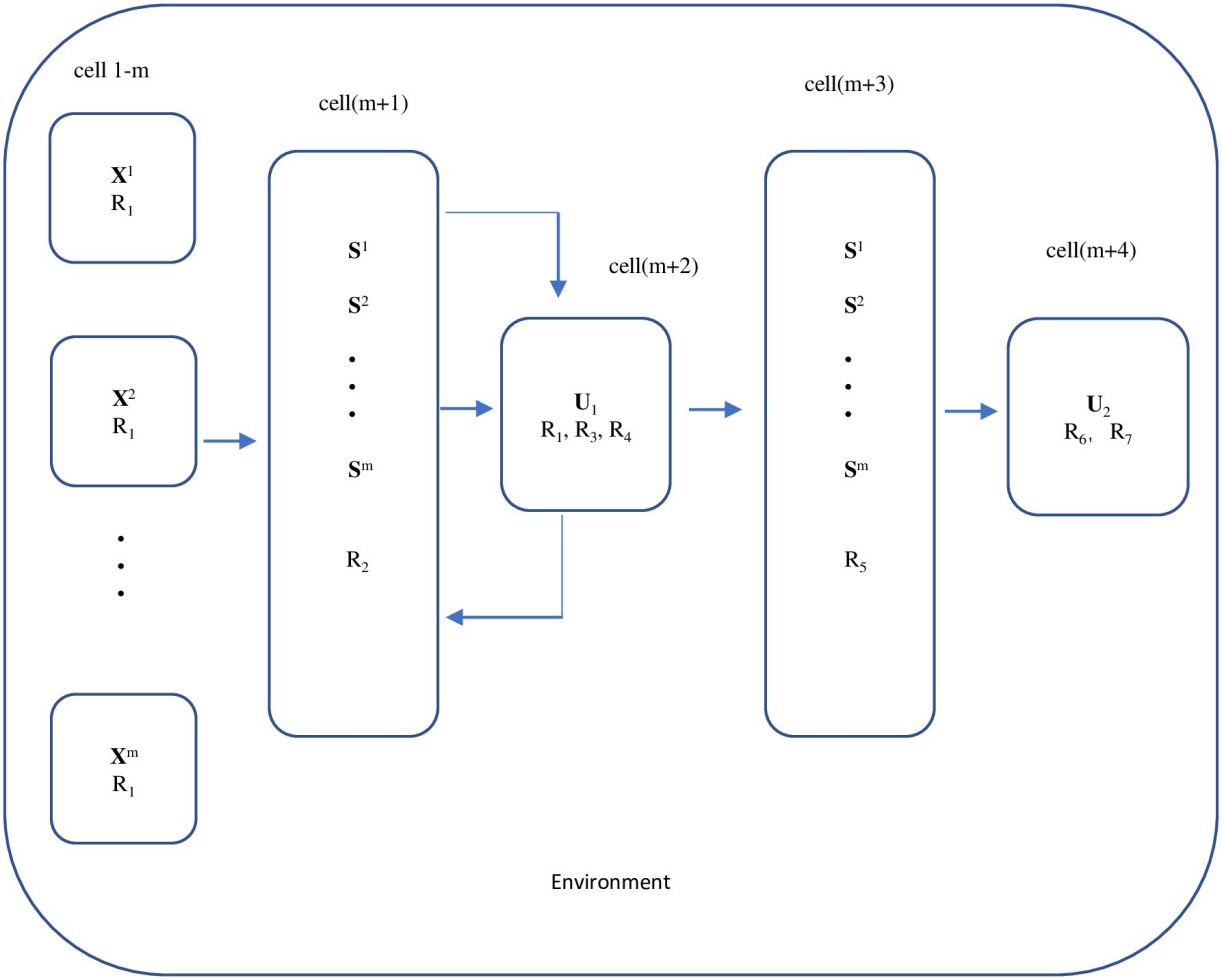
The RMVCP algorithm process in the framework of the tissue-like P system.

In the above-mentioned multi-view clustering algorithms, the weight distribution of each view and the weakening of noise data have not been effectively processed. Therefore, inspired by multi-view subspace clustering and graph-based multi-view clustering, in order to more effectively assign the weight of each view, Reweighted multi-view clustering with tissue-like P system (RMVCP) algorithm is proposed in this paper. RMVCP performs two fusion operations on each view. In the first fusion process, the self-representation matrix of each view is first constructed by the self-representation method, which can also be regarded as the similarity matrix of each view. Then assign appropriate weights to each view to fuse the similarity matrix of each view into a unified matrix. This operation is an iterative operation. Finally, the updated unified matrix and the updated similarity matrix of each view are generated. In the second fusion operation, the updated similarity matrix of each view generated in the first operation is used as input, and the appropriate weights are assigned to each view again to generate the final matrix. At the same time, Constrained Laplacian Rank (CLR) [45] is applied to the final matrix to directly generate clustering results without additional clustering steps (such as K-means). In addition, in order to improve the computational efficiency of the RMVCP algorithm, the RMVCP algorithm is integrated with the tissue-like P system. Fig 1 shows the RMVCP process without tissue-like P system. Fig 2 shows the RMVCP algorithm process in the framework of the tissue-like P system.

In summary, the contributions of our work are listed as follows:

- In order to assign weights to each view more reasonably, all views will be merged twice. The two fusion operations are iterative processes, which can assign more reasonable weights to each view.
- Constrained Laplacian Rank (CLR) are imposed on the unified matrix after the second fusion. Therefore, the clustering results can be directly output without applying additional clustering algorithms, avoiding the suboptimal solution of the existing two-step method.
- The RMVCP algorithm is integrated with the tissue-like P system, and use the computational parallelism of the tissue-like P system to improve the computational efficiency of the algorithm.
- The RMVCP algorithm integrates multiple processes into one framework. Experiments on several datasets prove that the clustering performance of our algorithm is better than other state-of-the-art algorithms.

The rest of this paper is organized as follows. The related research on multi-view clustering and the basic definition of the tissue-like P system are introduced in Section 2; In Section 3, RMVCP method is proposed; Comparative experiments were conducted to verify the effectiveness of the RMVCP algorithm in Section 4; At the end of the paper, we conclude in Section 5 and point out what we can do in the future.

## 2. Related work

### 2.1 Multi-view clustering

Currently, the most researched multi-view clustering methods are multi-view subspace clustering and graph-based multi-view clustering. Both multi-view subspace clustering and graph-based multi-view clustering have good clustering performance. Our RMVCP algorithm is also inspired by these two clustering methods. Multi-view subspace clustering uses multiple low-dimensional subspaces to represent high-dimensional data. Wang et al. [46] proposed Exclusivity-Consistency Regularity Multi-view Subspace Clustering (ECMSC). Many methods focus on the fusion of multiple views, without considering the direct consistency and difference information of the views. ECMSC considers a kind of exclusive information between views, so as to achieve information complementarity, which is helpful to improve the clustering performance. With the study of the potential representation of the data, Zhang et al. [47] proposed Latent Multi-view Subspace Clustering (LMSC). LMSC explores a latent representation of multi-view data, and then constructs a subspace representation from the latent representation. Zhang et al. integrated these two processes into an algorithm framework, while also reducing the impact of noise. High-dimensional data has always been a challenge for multi-view clustering. In order to cluster high-dimensional data more effectively, Wang et al. [48] proposed Multi-view Subspace Clustering with Intactness-Aware Similarity (MSC_IAS). MSC_IAS reduces the data dimension while preserving the data information, integrates it into a complete space, and constructs the similarity matrix. Then apply a clustering algorithm to the similarity matrix. This method can effectively process high-dimensional data. In order to more efficiently use the information across multiple views, Kang et al. [41] proposed Multi-graph Fusion for Multi-view Spectral Clustering (GFSC). GFSC can explore heterogeneous information between views, construct a similarity matrix with a self-representation method, and perform views fusion and spectral clustering at the same time. The noise information in the data greatly affects the clustering performance. In order to be able to reduce the noise information, Zhang et al. [42] proposed Consensus One-step Multi-view Subspace Clustering (COMVSC), COMVSC optimally integrates discriminative partition-level information, which can effectively reduce the impact of noise information. These state-of-the-art algorithms show good clustering performance, but the common defect is that only one fusion operation is performed on each view.

The graph-based multi-view clustering method first constructs the similarity matrix of each view, then merges each view into a unified matrix, and finally applies additional clustering algorithms or other methods to the unified matrix to obtain the clustering results. The construction of the similarity graph of each view is a very important step. Many scholars have proposed some methods for constructing similarity graphs, such as k-nearest neighbor algorithm(k-NN), Clustering with Adaptive Neighbors (CAN) [12], etc. On the other hand, the method of fusing each similarity graph is also very important. But similarly, the existing multi-view graph clustering method only merges each view once, so it does not achieve good clustering performance. Nie et al. [49] proposed the Parameter-Free Auto-Weighted Multiple Graph Learning (AMGL). AMGL solves the problem of multiple parameters in the fusion process, and automatically assigns the weight of each view on the basis of modifying the traditional spectral clustering method. Graph-based multi-view clustering methods need to apply additional clustering algorithms to obtain clustering results, and the two-step operation will affect the clustering performance. Nie et al. [50] proposed Self-weighted Multiview Clustering (SwMC). SwMC automatically assigns weights to each view without prior knowledge. At the same time, the clustering results are directly obtained without additional clustering algorithms. In addition, the quality of the similarity graph is affected by noisy data, which in turn affects the clustering results. Nie et al. [51] proposed Multi-View Clustering and Semi-Supervised Classification with Adaptive Neighbours (MLAN). MLAN obtains the final graph for clustering by learning the local manifold structure to alleviate the noise problem. Wang et al. [37] proposed GMC: Graph-Based Multi-View Clustering (GMC). GMC jointly builds multiple view graphs and fusion graph, and automatically assign weights to each view. Obviously, these state-of-the-art multi-view graph clustering algorithms only perform one fusion operation.

### 2.2 Tissue-like P system

The tissue-like P system is similar to a graph structure. In the tissue-like P system, each cell and environment are equivalent to the nodes of the graph, and the communication channels between cell to cell and cell to environment are equivalent to the edge of the graph. The calculation process of the tissue-like P system is to perform calculation operations in cells through rules, and then apply certain rules to transfer objects between cells and cells or between cells and the environment through communication channels. The basic definition of the tissue-like P system is as follows:

$$\begin{array}{c} \Pi =(O,K,\omega_{1},\ldots ,\omega_{m},E,ch,{\left(s_{\left(i,j\right)}\right)}_{(i,j)\in ch},{\left(R_{\left(i,j\right)}\right)}_{(i,j)\in ch},i_{0}) \end{array}$$ (2)

(1) O represents a finite multiset of objects;

(2) K represents the states of the alphabet;

(3) *ω*_*i*_, 1 ≤ *i* ≤ *m* represents the finite multiset of objects in the initial state of cells 1, …,m;

(4) *E* ⊆ *O* represents a copy of any number of symbolic objects in the environment;

(5) *ch* ⊆ {(*i*, *j*)|*i*, *j* ∈ {0, 1, …, *m*}, *i* ≠ *j*} represents the communication channel between cells and cells and between cells and the environment;

(6) *s*_(*i*, *j*)_ is the initial state of the channel (*i*, *j*);

(7) *R*_(*i*, *j*)_ is a finite co/inverse transportation rule of the form (*s*, *x*/*y*, *s*′), where *s*, *s*′ ∈ *K*, *x*, *y* ∈ O*;

(8) *i*_0_ ∈ {1, …, *m*}is the output cell.

## 3. Reweighted multi-view clustering with tissue-like P system (RMVCP)

Different from exploring the local information of data, the exploration of global information of data can better grasp the relationship between data points, which is the motivation for us to use the self-representation method to construct the similarity matrix of each view. Moreover, the quality of each view is uneven, and it is undesirable to treat each view equally. This prompted us to assign weight to each view. Nevertheless, twice weighting each view is necessary to improve clustering accuracy. In addition, the improved multi-view clustering algorithm is combined with tissue-like P system to improve the computational efficiency of the algorithm, which is due to the parallel computing ability of tissue-like P system. Therefore, we propose Reweighted multi-view clustering with tissue-like P system (RMVCP) in this paper, where RMVCP-1 refers to the first weight allocation process and RMVCP-2 is the second weight allocation for each view.

### 3.1 The first fusion process of the RMVCP model (RMVCP-1)

In the RMVCP-1 process, the similarity matrix of each view is constructed by a self-representation method [41]. Self-representation method treats each data point as a linear combination of the data itself. Given data matrix **X** ∈ *R*^d×n^. The similarity matrix **S** can be obtained by solving:

$$\begin{array}{c} \min_{S}∥X-XS{∥}_{F}^{2}+\alpha ∥S{∥}_{F}^{2}\;s.t.\;\;S\geq 0 \end{array}$$ (2)

where *α* is a trade-off parameter. Then we extend it to multi-view clustering:

$$\begin{array}{c} \min_{S^{v}}\sum_{v=1}^{m}∥X^{v}-X^{v}S^{v}{∥}_{F}^{2}+\alpha ∥S^{v}{∥}_{F}^{2}\;s.t.\;\;S^{v}\geq 0 \end{array}$$ (3)

where m is the number of the views. The similarity matrix of each view obtained by this formula reflects different aspects of the original data. On the basis of this formula, the construction of the unified matrix **U**_1_ is as follows:

$$\begin{array}{c} U_{1}=\frac{\sum_{v=1}^{m}S^{v}}{m} \end{array}$$ (4)

Obviously, the construction of the unified matrix is simply adding the similarity matrix of each view and dividing by the number of views without considering the weight of each view. This will lead to poor clustering performance. Therefore, we calculate the weight of each view in the process of graph fusion. The formula is as follows:

$$\begin{array}{c} \min_{U_{1}}\sum_{v=1}^{m}w_{v}∥S^{v}-U_{1}{∥}_{F}^{2} \end{array}$$ (5)

where *w*_*v*_ is the weight of each view. In this way, the unified matrix can better reflect the characteristics of the data with good views are given large weights and bad views are given small weights. The expression of weight is:

$$\begin{array}{c} w_{v}=\frac{1}{2∥S^{v}-U_{1}{∥}_{F}} \end{array}$$ (6)

Then the goal formula is proposed by combining Eqs 3 and 5:

$$\begin{array}{c} \begin{array}{c} \min_{S^{v},U_{1},F}\sum_{v=1}^{m}∥X^{v}-X^{v}S^{v}{∥}_{F}^{2}+\alpha ∥S^{v}{∥}_{F}^{2}+\beta w_{v}∥S^{v}-U_{1}{∥}_{F}^{2}\\ s.t.\;\;S^{v}\geq 0 \end{array} \end{array}$$ (7)

By solving Eq 7, we can learn the similarity matrix of each view and the final unified matrix after weighting by an iterative algorithm. Finally, the unified matrix is fed to the spectral clustering algorithm.

For clustering, an ideal situation is that the number of connected components of the similarity matrix is equal to the number of clusters. When this situation is met, that is, the number of connected components of the similarity matrix is equal to k, the data point can be exactly divided into k clusters. So here we introduce Theorem 1 [52, 53].

**Theorem 1**. *The multiplicity of the eigenvalue 0 of the Laplacian matrix of the similarity matrix is equal to the number of connected components in the graph of the similarity matrix*.

According to Theorem 1, we know that when the number of eigenvalues 0 of the similarity matrix is k, the number of connected components is exactly k. From the Ky Fan’s Theory [54], we get the final expression of Eq 7 as follows(The specific process is shown in the RMVCP-2 process):

$$\begin{array}{c} \begin{array}{c} \min_{S^{v},U_{1},F}\sum_{v=1}^{m}∥X^{v}-X^{v}S^{v}{∥}_{F}^{2}+\alpha ∥S^{v}{∥}_{F}^{2}+\beta w_{v}∥S^{v}-U_{1}{∥}_{F}^{2}+\gamma Tr\left(F_{U_{1}}^{T}L_{U_{1}}F_{U_{1}}\right)\\ s.t.\;\;S^{v}\geq 0,F_{U_{1}}^{T}F_{U_{1}}=I \end{array} \end{array}$$ (8)

where $F_{U_{1}}$ is the spectral embedding matrix, $L_{U_{1}}$ is the Laplacian matrix of the unified matrix, and *α*, *β*, *γ* are regularization parameters.

Next, we optimize Eq 8:

We optimize each variable through an iterative method.

**Updating S**_*v*_ **when** $F_{U_{1}}$ **and U**_1_ **are fixed. So** Eq 7 **becomes**:

$$\begin{array}{c} \min_{S^{v}}\sum_{v=1}^{m}∥X^{v}-X^{v}S^{v}{∥}_{F}^{2}+\alpha ∥S^{v}{∥}_{F}^{2}+\beta w_{v}∥S^{v}-U_{1}{∥}_{F}^{2} \end{array}$$ (9)

It can be seen from Eq 9 that each view is independent. So, we only consider one view at a time. In order to update **S**_*v*_, we perform the derivative operation on Eq 9 to obtain:

$$\begin{array}{c} {(\text{Eq}\;9)}^{•}=-2{\left(X^{v}\right)}^{T}\left(X^{v}-X^{v}S^{v}\right)+2\alpha S^{v}+2\beta w_{v}\left(S^{v}-U_{1}\right) \end{array}$$ (10)

Then we make Eq 10 equal to zero and we get:

$$\begin{array}{c} S^{v}={\left({\left(X^{v}\right)}^{T}X^{v}+\alpha I+\beta w_{v}I\right)}^{-1}\left(\beta w_{v}U_{1}+{\left(X^{v}\right)}^{T}X^{v}\right) \end{array}$$ (11)

where **I** is the identity matrix.

**Updating U_1_ when** $F_{U_{1}}$ **and S**_*v*_ **are fixed.** We obtain:

$$\begin{array}{c} \min_{S}\sum_{v=1}^{m}\beta w_{v}∥S^{v}-U_{1}∥+\gamma Tr\left(F_{U_{1}}^{T}L_{U_{1}}F_{U_{1}}\right) \end{array}$$ (12)

After deriving [41], we can obtain:

$$\begin{array}{c} U_{1}\left(:,i\right)=\frac{\sum {}_{{}_{v}}w_{v}S^{v}\left(:,i\right)-\frac{\gamma q_{i}}{4\beta }}{\sum {}_{{}_{v}}w_{v}} \end{array}$$ (13)

where **q**_*i*_ ∈ *R*^*n*×1^ with the j-th entry $q_{ij}=∥F_{U_{1}i,:}-F_{U_{1}j,:}{∥}^{2}$.

**Updating** $F_{U_{1}}$ **when S**_*v*_ **and U**_1_ **are fixed.** We need to solve the following problems:

$$\begin{array}{c} \min_{F_{U_{1}}}Tr\left(F_{U_{1}}^{T}L_{U_{1}}F_{U_{1}}\right)\;s.t.\;\;{F_{U_{1}}}^{T}F_{U_{1}}=I \end{array}$$ (14)

$F_{U_{1}}$ is composed of the eigenvectors corresponding to the first k smallest eigenvalues of the Laplacian matrix. We terminate algorithm 1 when the number of iterations is greater than 200 or the relative change of **U**_1_ is less than 0.001. So far, we have calculated the similarity matrix of each view that will be input to the next algorithm after the first iteration. The RMVCP-1 process is summarized in Algorithm 1.

**Algorithm 1** RMVCP-1

**Input:** Data matrices: **X**^1^,…, **X**^*m*^, parameters *α* > 0, *β* > 0, *γ* > 0.

**output:** Similarity matrices: **S**^1^,…, **S**^*m*^, Unified matrix **U**_1_, $F_{U_{1}}$.

**Initialize:** Random matrices **U**_1_ and $F_{U_{1}}$, *w*_*v*_ = 1/*m*.

**repeat**

 1: Update **S**_*v*_ by Eq 11 for each view.

 2: For each element $S_{ij}^{v}=\text{max}\left(S_{ij}^{v},0\right)$.

 3: Update **U**_1_ by Eq 13.

 4: Update $F_{U_{1}}$ by Eq 14.

 5: Update *w*_*v*_ by Eq 6.

**until** stopping criterion is met.

### 3.2 The second fusion process of the RMVCP model (RMVCP-2)

RMVCP-1 continuously updates **S**^1^,…, **S**^*m*^ in an iterative manner until the algorithm converges. We perform another fusion process again, and constantly update **S**^1^,…, **S**^*m*^ so that the similarity matrix **S**^1^,…, **S**^*m*^ can better represent the characteristics of each view. We use the updated **S**^1^,…, **S**^*m*^ obtained from RMVCP-1 as the input of RMVCP-2. The objective function [50] is:

$$\begin{array}{c} \min_{u_{2ij}\geq 0,u_{2i}1_{n}=1}\sum_{v=1}^{m}\alpha^{\left(v\right)}∥U_{2}-S^{\left(v\right)}{∥}_{F}^{2} \end{array}$$ (15)

where **U**_2_ is the unified matrix, and *α*^(*v*)^ is the weight of the v-th view:

$$\begin{array}{c} \alpha^{\left(v\right)}=\frac{1}{2∥U_{2}-S^{\left(v\right)}{∥}_{F}} \end{array}$$ (16)

In this case, the unified matrix **U**_2_ is obtained after fusion, and additional clustering algorithms are applied to the unified matrix **U**_2_, which will affect the final clustering performance. Here we introduce the Constrained Laplacian Rank (CLR) method to avoid additional clustering algorithms and directly output the clustering results.

It can be seen from Theorem 1 that when the rank of the Laplacian matrix of the unified matrix **U**_2_ is $L_{U_{2}}=n-c$, where c is the multiplicity of the eigenvalue 0 of $L_{U_{2}}$, the data points can be directly divided into c clusters. So, Eq 15 becomes:

$$\begin{array}{c} \min_{u_{2ij}\geq 0,u_{2i}1_{n}=1,rank\left(L_{U_{2}}\right)=n-c}\sum_{v=1}^{m}\alpha^{\left(v\right)}∥U_{2}-S^{\left(v\right)}{∥}_{F}^{2} \end{array}$$ (17)

It is very difficult to solve Eq 17. Let $\xi_{i}\left(L_{U_{2}}\right)$ denote the i-th smallest eigenvalue of $L_{U_{2}}.\xi_{i}\left(L_{U_{2}}\right)\geq 0$ since $L_{U_{2}}$ is positive semi-definite. Then, $rank(L_{U_{2}})=n-c$ can be achieved if $\sum_{i=1}^{c}\xi_{i}\left(L_{U_{2}}\right)=0$. From the Ky Fan’s Theory, we know:

$$\begin{array}{c} \sum_{i=1}^{c}\xi_{i}\left(L_{U_{2}}\right)=\min_{F\in R^{n\times c},{F_{U_{2}}}^{T}F_{U_{2}}=I}Tr\left(F_{U_{2}}^{T}L_{U_{2}}F_{U_{2}}\right) \end{array}$$ (18)

So, Eq 17 becomes:

$$\begin{array}{c} \begin{array}{c} \min_{U_{2},F_{U_{2}}}\sum_{v=1}^{m}\alpha^{\left(v\right)}∥U_{2}-S^{\left(v\right)}{∥}_{F}^{2}+2\lambda Tr\left(F_{U_{2}}^{T}L_{U_{2}}F_{U_{2}}\right)\\ s.t.\;\;u_{2ij}\geq 0,u_{i}1_{n}=1,F_{U_{2}}\in R^{n\times c},F_{U_{2}}^{T}F_{U_{2}}=I \end{array} \end{array}$$ (19)

The optimization of this formula is as follows:

**Updating** $F_{U_{2}}$ **when U**_2_, *α*^(*v*)^ **is fixed.** Eq 18 becomes:

$$\begin{array}{c} \min_{F_{U_{2}}\in R^{n\times c},F_{U_{2}}^{T}F_{U_{2}}=I}Tr\left(F_{U_{2}}^{T}L_{U_{2}}F_{U_{2}}\right) \end{array}$$ (20)

$F_{U_{2}}$ is formed by the c eigenvectors corresponding to the first c smallest eigenvalues of $L_{U_{2}}$.

**Updating U**_2_ **when** $F_{U_{2}}$ , *α*^(*v*)^ **is fixed.** Eq 19 becomes:

$$\begin{array}{c} \min_{u_{2ij}\geq 0,u_{2i}1_{n}=1}\sum_{v=1}^{m}\alpha^{v}\sum_{i,j=1}^{n}{\left(u_{2ij}-s_{ij}^{\left(v\right)}\right)}^{2}+\lambda \sum_{i,j=1}^{n}∥f_{U_{2}i}-f_{U_{2}j}{∥}_{2}^{2}u_{2ij} \end{array}$$ (21)

It is obvious that Eq 21 is independent for each i, so we consider each i separately:

$$\begin{array}{c} \min_{u_{2ij}\geq 0,u_{2i}1_{n}=1}\sum_{j=1}^{n}\sum_{v=1}^{m}\alpha^{\left(v\right)}{\left(u_{2ij}-s_{ij}^{\left(v\right)}\right)}^{2}+\lambda \sum_{j=1}^{n}∥f_{U_{2}i}-f_{U_{2}j}{∥}_{2}^{2}u_{2ij} \end{array}$$ (22)

We denote $r_{ij}=∥f_{U_{2}i}-f_{U_{2}j}{∥}_{2}^{2}$ for the purpose of avoiding Eq 22 that are too complicated. So, Eq 22 can be written in vector form as:

$$\begin{array}{c} \min_{u_{2i}\geq 0,u_{2i}1_{n}=1}∥u_{2i}-\left(\sum_{v=1}^{m}\alpha^{\left(v\right)}s_{i}^{\left(v\right)}-\frac{\lambda }{2}r_{i}\right)/\sum_{v=1}^{m}\alpha^{\left(v\right)}{∥}_{2}^{2} \end{array}$$ (23)

The problem can be solved by an iterative algorithm. Algorithm 1 shows the process of the second fusion.

**Algorithm 2** RMVCP-2

**Input:** **S**^1^,…, **S**^*m*^ ∈ *R*^*n*×*n*^ obtained by algorithm 1, number of clusters c.

**output:** **U**_2_ ∈ *R*^*n*×*n*^ with c connected components.

**Initialize:** the weight for each view $\alpha^{\left(v\right)}=\frac{1}{m}$, $F_{U_{2}}$ is composed of the eigenvectors corresponding to the first c smallest eigenvalues of $L_{U_{2}}=D_{U_{2}}-\frac{U_{2}^{T}+U_{2}}{2}$.

**repeat**

 **repeat**

  1: Calculate and update **U**_2_ by Eq 23.

  2: Update $F_{U_{2}}$ by Eq 20

 **untill** converge Update *α*^(*v*)^ by Eq 16.

**untill** converge

RMVCP takes the similarity matrix **S**^1^,…, **S**^*m*^ of each view generated by the iterative update process of Algorithm 1 as input into Algorithm 1, and can directly output the clustering results.

### 3.3 Initial configuration of the tissue-like P system

In this paper, in order to improve the computational efficiency of the RMVCP algorithm, we combine RMVCP with the tissue-like P system. We first set up the initial configuration of the tissue-like P system in this paper.

- cell i, 1 ≤ *i* ≤ *m*: Multiset of objects $\omega_{i}=X^{i},w_{i},F_{U_{1}},U_{1}$;
  *R*_1_: Rule *R*_1_ uses Eq 11 to generate **S**^*v*^, 1 ≤ *v* ≤ *m* and send it to the cell (m+1);
  *R*_10_: Rule *R*_10_ sends copies of *α*, *β*, *I* in the environment to cell m-1.
- cell (m+1): Multiset of objects *ω*_m+1_ = *w*_1_, …, *w_m_*, $F_{U_{1}}$;
  *R*_2_: Rule *R*_2_ uses Eq 13 to generate the updated $U_{1}$ and send it to the cell (m+2);
  *R*_20_: Rule *R*_20_ sends copies of *β* in the environment to the cell (m+1).
- cell (m+2): Multiset of objects *ω*_m+2_ = **X**^1^, …, **X**^*m*^ *w*_1_, …, *w*_*m*_;
  *R*_1_: Rule *R*_1_ uses Eq 11 to generate the updated **S**^*v*^, 1 ≤ *v* ≤ *m* and send it to the cell (m+1);
  *R*_10_: Rule *R*_10_ sends copies of *α*, *β*, *I* in the environment to cell m-1.
  *R*_3_: Rule *R*_3_ uses Eq 6 to generate the weight *w*_1_, …, *w*_*m*_ for each view;
  *R*_4_: Rule *R*_4_ uses Eq 14 to generate the updated object $F_{U_{1}}$;
  *R*_40_: Rule *R*_40_ sends the **S**^*v*^, 1 ≤ *v* ≤ *m* in the cell (m+2) to the cell (m+3). Rule *R*_40_ can only be triggered when the relative change of **U**_1_ is less than 0.001.
- cell (m+3): *ω*_m+3_ = *α*^1^, … *α*_m_, $F_{U_{2}}$;
  *R*_5_: Rule *R*_5_ uses Eq 26 to generate $U_{2}$ and send it to the cell (m+4). At the same time, rule *R*_5_ calculates the relative change of Eq 19.
- cell (m+4): *R*_6_: Rule *R*_6_ uses Eq 20 to generate the updated object $F_{U_{2}}$;
  *R*_7_: Rule *R*_7_ uses Eq 16 to generate updated object *α*^1^, …, *α*^*m*^;
- Environment:E=α,β,γ,I.

**Fig 3:**
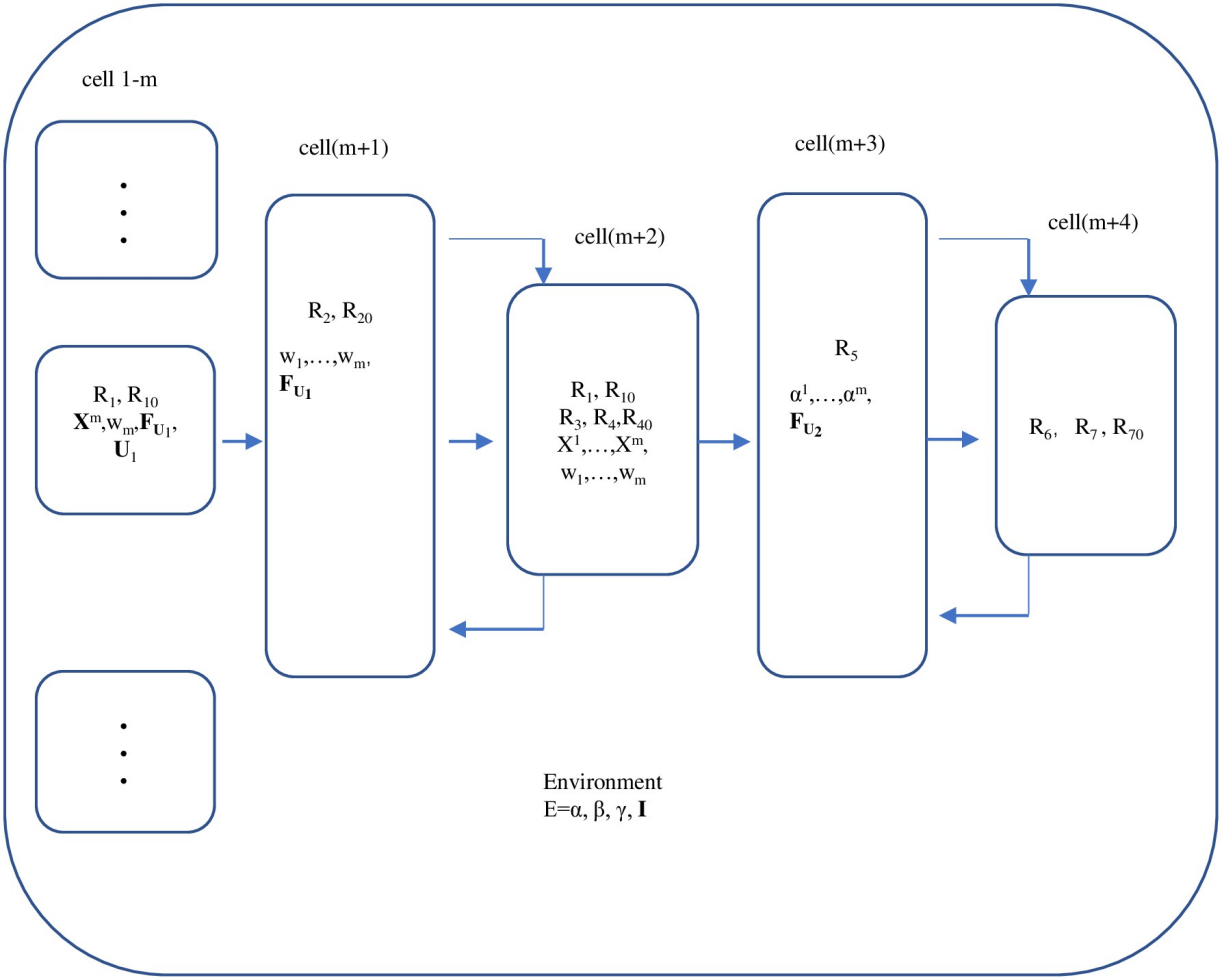
The initial configuration of the tissue-like P system.

Fig 3 shows the initial configuration of the tissue-like P system.

### 3.4 Computational process

- Step 1: In cells 1 to m, we first simultaneously apply the rule *R*_10_ to transfer the copies of *α*, *β*, **I** in the environment to the membrane, and then apply the rule *R*_1_ to generate **S**^*v*^, 1 ≤ *v* ≤ *m* and send it to the cell (m+1).
- Step 2: In cell (m+1), the rule *R*_20_ is applied to transfer the copy of *β* in the environment to the membrane, and then the rule *R*_2_ is applied to generate $U_{1}$ and send it to cell (m+2).
- Step 3: In this step, first apply the rule *R*_3_ to generate the updated weight *w*_1_, …, *w*_*m*_, and then the rule *R*_4_ is applied to generate the updated $F_{U_{1}}$. Next the rule *R*_10_ is applied to transfer the copies of *α*, *β*, **I** in the environment to the cell (m+2), and then apply the rule *R*_1_ to produce the updated **S**^*v*^, 1 ≤ *v* ≤ *m* and send it to the cell (m+1). Then apply the rules in the cell (m+1) again.

Steps 2 to 3 are a cyclic process.

- Step 4: When the conditions of triggering rule *R*_40_ are met, rule *R*_40_ is triggered, and the updated **S**^*v*^, 1 ≤ *v* ≤ *m* is generated and sent to the cell (m+3). In the cell (m+3), after receiving the updated **S**^*v*^, 1 ≤ *v* ≤ *m* from the cell (m+2), the rule *R*_5_ is applied to generate **U**_2_ and send it to the cell (m+4).
- Step 5: When the cell (m+4) receives the **U**_2_ sent from the cell (m+3), the rules *R*_6_ and *R*_7_ are applied to generate updated $F_{U_{2}}$ and *α*^1^, …, *α*^*m*^, which are sent back to the cell (m+3). Then apply rule *R*_5_ in the cell (m+3) again.

Steps 4 to 5 are a cyclic process.

- Step 6(Termination of calculation): The calculation is terminated when the relative change of Eq 18 does not exceed 10^−8^. Then the updated **U_2_** is output. The specific process is shown by Algorithm 3.

**Algorithm 3** RMVCP

**Input:** Data matrices: **X**^1^, …, **X**^*m*^, parameters *α* > 0, *β* > 0, *γ* > 0, **I**.

**output:** Unified matrix **U**_1_, $F_{U_{1}}$, **U**_2_ ∈ *R*^*n*×*n*^ with c connected components, $F_{U_{2}}$.

**Initialize:** Random matrices **U**_1_ and $F_{U_{1}}$, *w*_*v*_ = 1/*m*, random *α*^*v*^, 1 ≤ *v* ≤ *m*.

1: cell 1-m: *R*_10_, *R*_1_: Generate **S**^*v*^, 1 ≤ *v* ≤ *m* and send it to the cell (m+1);

**repeat**

 2: cell (m+1): *R*_20_, *R*_2_: The updated $U_{1}$ is generated and sent to the cell (m+2);

 3: cell (m+2): *R*_3_: The updated *w*_1_, …, *w*_*m*_ are generated and sent to the cell (m+1);

  *R*_4_: The updated $F_{U_{1}}$ is generated and sent to the cell (m+1);

  *R*_10_, *R*_1_: The updated **S**^*v*^, 1 ≤ *v* ≤ *m* are generated and sent to the cell (m+1).

**untill** Rule *R*_40_ is triggered, and the updated **S**^*v*^, 1 ≤ *v* ≤ *m* are generated and sent to the cell (m+3).

**repeat**

 4: cell (m+3): *R*_5_: The updated $U_{1}$ is generated and sent to the cell (m+4);

 5: cell (m+4): *R*_6_: $F_{U_{2}}\to$ cell (m+3);

  *R*_7_: The updated *α*^1^, …, *α*^*m*^ are generated and sent to the cell (m+3);

**untill** The condition for termination of calculation is met. Output **U**_1_.

### 3.5 Convergence analysis of RMVCP-2

In this section we prove the convergence of RMVCP-2.

**Lemma 1**. *For any positive numbers a and b, inequality* 23 *holds*:

$$\begin{array}{c} a-\frac{a^{2}}{2b}\leq b-\frac{b^{2}}{2b} \end{array}$$ (24)

*Proof*. We use ${\tilde{U}}_{2}$ to represent the updated **U_2_** after each iteration. After the first iteration of the loop, we get:

$$\begin{array}{c} {\tilde{U}}_{2}=\underset{u_{2ij}\geq 0,u_{2i}1_{n}=1,rank\left(L_{U_{2}}\right)=n-c}{\text{arg}\;\text{min}}\sum_{v=1}^{m}\alpha^{\left(v\right)}∥U_{2}-S^{\left(v\right)}{∥}_{F}^{2} \end{array}$$ (25)

Because of $\alpha^{\left(v\right)}=\frac{1}{2∥U_{2}-S^{\left(v\right)}{∥}_{F}}$, we obtain:

$$\begin{array}{c} \sum_{v=1}^{m}\frac{∥{\tilde{U}}_{2}-S^{\left(v\right)}{∥}_{F}^{2}}{2∥U_{2}-S^{\left(v\right)}{∥}_{F}}\leq \sum_{v=1}^{m}\frac{∥U_{2}-S^{\left(v\right)}{∥}_{F}^{2}}{2∥U_{2}-S^{\left(v\right)}{∥}_{F}} \end{array}$$ (26)

From Lemma 1, we obtain:

$$\begin{array}{c} \begin{array}{c} \sum_{v=1}^{m}∥{\tilde{U}}_{2}-S^{\left(v\right)}{∥}_{F}-\sum_{v=1}^{m}\frac{∥{\tilde{U}}_{2}-S^{\left(v\right)}{∥}_{F}^{2}}{2∥U_{2}-S^{\left(v\right)}{∥}_{F}}\\ \leq \sum_{v=1}^{m}∥U_{2}-S^{\left(v\right)}{∥}_{F}-\sum_{v=1}^{m}\frac{∥U_{2}-S^{\left(v\right)}{∥}_{F}^{2}}{2∥U_{2}-S^{\left(v\right)}{∥}_{F}} \end{array} \end{array}$$ (27)

After deduction, we obtain:

$$\begin{array}{c} \sum_{v=1}^{m}∥{\tilde{U}}_{2}-S^{\left(v\right)}{∥}_{F}\leq \sum_{v=1}^{m}∥U_{2}-S^{\left(v\right)}{∥}_{F} \end{array}$$ (28)

Therefore, it can be seen that the value of the objective function of each iteration will decrease, and finally meet the KKT condition of the objective function, and converge to the local optimal solution.

### 3.6 Complexity analysis

RMVCP-1:

The complexity of RMVCP-1 mainly comes from the update of **S**^(*v*)^ and $F_{U_{1}}$. When updating **S**^(*v*)^, it costs O(*n*^3^) due to matrix multiplication and matrix inversion. In the process of updating $F_{U_{1}}$, the operation of singular value decomposition takes O(*n*^3^).

RMVCP-2:

The complexity of RMVCP-2 mainly comes from the process of updating the weights *α*^(*v*)^ and $F_{U_{2}}$. The complexity of updating weight *α*^(*v*)^ is O(*mn*^2^). When updating $F_{U_{2}}$, it is necessary to calculate the eigenvector of the Laplacian matrix of **U**_2_, so the complexity of updating $F_{U_{2}}$ is O(*cn*^2^).

## 4. Experiments

### 4.1 Datasets

In order to verify the clustering performance of our proposed RMVCP algorithm, we conduct comparative experiments on five public datasets. The five datasets include ORL, MSRC, HW, Yale, Wikipedia Article. The specific information of the five datasets is as follows:

- ORL: ORL [55] is an image dataset, which contains 400 images from 40 people. Each person has 10 different images. ORL has four views, namely GIST (512), LBP (59), HOG (864) and CENTRIST (254) (the dimensions of each view are in parentheses).
- MSRC: MSRC [56] is an image dataset. It contains 210 samples of 7 types. The 7 categories are bicycle, tree, car, airplane, building, cow, face. There are 30 images in each category. There are six views in MSRC, namely CENTRIST (1302), CMT (48), GIST (512), HOG (100), LBP (256), SIFT (210).
- HW: HW [57] is an image dataset. It contains 2000 images in 10 categories. These 10 categories respectively show one of the 10 numbers “0–9”. There are 200 images in each category. HW has 6 views, namely FAC (216), FOU (76), KAR (64), MOR (6), PIX (240), ZER (47).
- Yale: The Yale dataset [56] is an image dataset. It contains 165 samples in 15 categories. Each category shows a different person, and each person has 11 different states, wearing glasses and not wearing glasses, and so on. Yale has three views, namely Intensity (4096), LBP (3304), Gabor (6750).
- Wikipedia Article: Wikipedia Article [58] is a dataset composed of featured articles selected from Wikipedia. It contains 693 samples in 10 categories. There are two views in Wikipedia Article, with feature dimensions of 128 and 10 respectively.

The specific information of these five datasets is shown in Table 1, where d1, d2, d3, d4, d5, d6 is the number of features in each view, n is the number of samples, and c is the number of clusters.

**Table 1:** The specific details of the five datasets ORL, MSRC, HW, Yale, Wikipedia Article.

	d1	d2	d3	d4	d5	d6	n	c
ORL	512	59	864	254	-	-	400	40
MSRC	1302	48	512	100	256	210	210	7
HW	216	76	64	6	240	47	2000	10
Yale	4096	3304	6750	-	-	-	165	15
Wikipedia Article	128	10	-	-	-	-	693	10

### 4.2 Comparison algorithms and evaluation indicators

In this paper, in order to prove the effectiveness of our proposed RMVCP algorithm, we compare the RMVCP algorithm with some other state-of-the-art algorithms. These algorithms include single-view spectral clustering (SC), connected feature methods (CF), Auto-weighted Multiple Graph Learning (AMGL) [49], One-step Multi-view Spectral Clustering (OMSC) [34], Multi-view Concept Clustering (MVCC) [59], Multi-View Clustering via Deep Matrix Factorization (MVC-DMF) [60], Deep Matrix Factorization based Solution (DMFClusts) [61], Binary Multi-view Clustering (BMVC) [62], Multi-graph Fusion for Multi-view Spectral Clustering (GFSC) [41], Multi-View Clustering in Latent Embedding Space (MCLES) [63], Multi-view clustering via deep concept factorization (MCDCF) [64].

In order to verify the clustering performance of each algorithm, the evaluation criteria we adopt in this paper are **Accuracy (Acc), Normalized Mutual Information (NMI), Purity** [65]. The calculation methods of these performance metrics are as follows:

**Acc** is used to verify whether the obtained label is consistent with the real label provided by the data:

$$\begin{array}{c} \text{Acc}=\frac{\sum_{i=1}^{n}\tau (z^{i},map\left(o_{i}\right))}{N} \end{array}$$

where *z*^*i*^ is the label after clustering, *o*_*i*_ is the true label, n is the total number of data points, *τ* is the indicator function.

**NMI**: First define *A* and *B* as two random variables, *H*(*A*) and *H*(*B*) are their corresponding entropy respectively, then use the following formula to calculate NMI:

$$\begin{array}{c} NMI\left(A,B\right)=\frac{I(A,B)}{\sqrt{H(A)H(B)}} \end{array}$$

where *I*(*A*, *B*) represents the mutual information between *A* and *B*. The larger the value, the better the performance.

**Purity**: Purity is defined as the proportion of documents that are correctly clustered to the total documents. The formula is as follows:

$$\begin{array}{c} purity=\frac{1}{N}\sum_{i=1}^{k}\text{max}|b_{i}\cap g_{j}| \end{array}$$

*b*_*i*_ represents the i-th cluster and *g*_*j*_ represents the classification that has the maximum count for cluster *b*_*i*_.

### 4.3 Evaluation of experimental results

**Fig 4:**
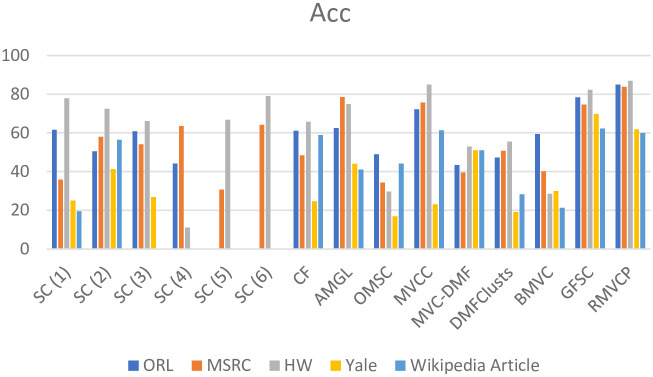
A histogram comparison of the Acc results of all algorithms on the five datasets.

**Fig 5:**
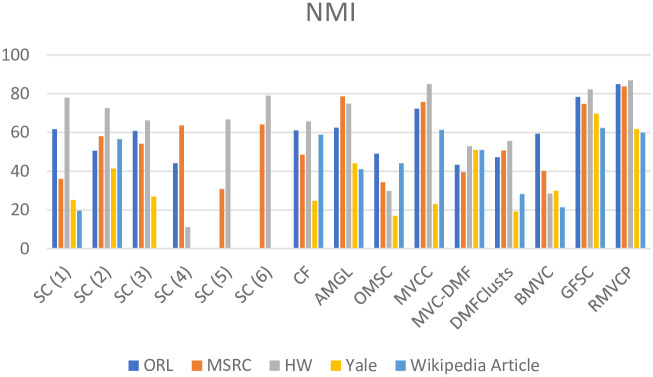
A histogram comparison of the NMI results of all algorithms on the five datasets.

**Fig 6:**
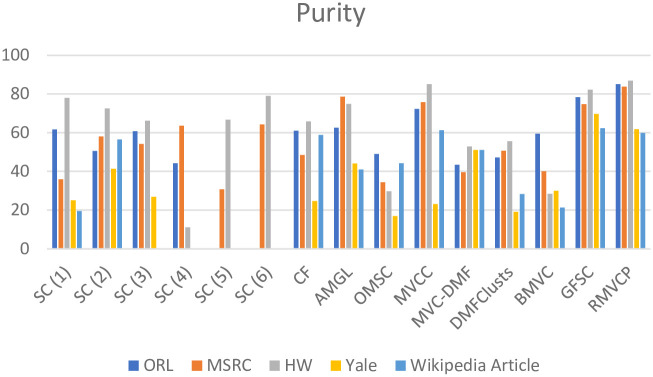
A histogram comparison of the Purity results of all algorithms on the five datasets.

We conduct experiments on five datasets ORL, MSRC, HW, Yale and Wikipedia Article, and the compared algorithms are SC, CF, AMGL, OMSC, MVCC, MVC-DMF, DMFClusts, BMVC, GFSC, MCLES, MCDCF. Tables 2–4 respectively shows the comparison of the Acc, NMI, and Purity results of the RMVCP algorithm and other algorithms on the five data sets. The best results are highlighted in bold and the second-best results are underlined. Figs 4–6 shows the histogram comparison of the Acc, NMI, and Purity results of all algorithms on the five data sets.

- It can be seen from the experimental results that compared to multi-view clustering, the clustering performance of single-view clustering is worse than that of multi-view clustering. In these five data sets, the clustering performance of the spectral clustering algorithm for each view is not very satisfactory. From the Acc results, for ORL, MSRC, HW, Yale, Wikipedia Article data sets, the best spectral clustering results are 25.87%, 22.66%, 7.26%, 24.02%, 2.78% lower than RMVCP, respectively. This fully shows that the RMVCP algorithm is better than the single-view spectral clustering algorithm.
- For the feature connection method, all the features are connected together and single-view spectral clustering is performed on them. This method simply superimposes the features together, and the Acc results on the five data sets are 26,3%, 36.85%, 20.33%, 38.62%, 1.24% lower than the RMVCP algorithm respectively. This fully illustrates the importance of assigning weights to views.
- Compared with these multi-view clustering algorithms, in general, the RMVCP algorithm is better than other multi-view clustering algorithms. From the Acc results, the MVCC algorithm is second only to the RMVCP algorithm, which shows that the multi-view conceptual clustering method has good clustering performance in the multi-view clustering, and the RMVCP algorithm is superior to the conceptual clustering method.
- The GFSC algorithm uses a self-representation method to generate the similarity matrix of each view, without performing the second fusion, and finally uses an additional spectral clustering step to generate the final clustering result. It can be seen from the results that the clustering performance of the GFSC algorithm is worse than that of the RMVCP algorithm, which indicates that the secondary fusion can allocate the weight of views more reasonably and reduce the influence of noise information. At the same time, in terms of accuracy, the performance of directly generating clustering results is better than using additional clustering steps.
- AMGL is a self-weighted graph learning method with good clustering performance, but only one weight assignment is performed in the clustering process. It can be seen from the results that the RMVCP algorithm is superior to the AMGL algorithm in all aspects, which illustrates the importance of the secondary distribution of weights.
- MCLES searches for the potential embedding space of data to explore the global information of data. Concept decomposition and deep learning are applied to multi-view clustering by MCDCF. The experimental results reveal that the running time of MCLES and MCDCF on the HW dataset with 2000 data points is more than an hour, which indicates that the two algorithms cannot deal with a slightly larger dataset, and RMVCP can deal with this kind of dataset.

**Table 2:** Comparison of algorithms on five datasets for Acc.

	ORL	MSRC	HW	Yale	Wikipedia Article
SC (1)	53.28	33.00	73.44	24.14	17.36
SC (2)	44.93	56.00	71.23	37.80	52.63
SC (3)	54.63	50.00	63.49	25.25	-
SC (4)	39.60	57.86	10.84	-	-
SC (5)	-	28.33	63.44	-	-
SC (6)	-	60.67	75.49	-	-
CF	54.20	46.48	62.42	23.20	54.17
AMGL	54.00	72.86	71.13	41.98	38.23
OMSC	39.00	31.43	12.35	16.15	22.08
MVCC	67.75	75.71	**84.11**	21.77	**58.66**
MVC-DMF	41.16	36.27	42.07	47.23	43.72
DMFClusts	41.75	46.71	50.93	15.37	23.13
BMVC	56.93	39.90	27.18	29.25	19.29
GFSC	63.68	56.43	70.48	55.88	49.61
MCLES	76.25	74.29	~ 1	6.67	54.11
MCDCF	70.25	82.76	~ 1	59.94	45.80
RMVCP	**80.50**	**83.33**	82.75	**61.82**	55.41

^1^‘~’ indicates that the running time of the algorithm exceeds one hour, and the following is the same.

**Table 3:** Comparison of algorithms on five datasets for NMI.

	ORL	MSRC	HW	Yale	Wikipedia Article
SC (1)	74.14	21.46	77.62	18.12	5.33
SC (2)	67.28	46.17	72.12	37.62	49.81
SC (3)	74.31	43.45	63.05	20.64	-
SC (4)	57.15	58.23	1.06	-	-
SC (5)	-	15.14	67.03	-	-
SC (6)	-	52.19	80.67	-	-
CF	74.57	41.26	64.92	17.61	51.09
AMGL	75.91	73.20	78.69	43.61	26.60
OMSC	53.93	20.74	22.32	6.87	35.78
MVCC	84.35	65.25	78.81	11.92	51.49
MVC-DMF	60.81	19.55	46.48	45.51	44.75
DMFClusts	63.66	32.81	46.20	3.41	17.32
BMVC	73.91	21.23	13.96	21.75	7.84
GFSC	81.83	51.05	74.19	60.67	43.15
MCLES	88.58	70.40	~	0	46.90
MCDCF	85.30	76.06	~	64.22	34.82
RMVCP	**89.07**	**84.25**	**87.62**	**64.42**	**51.58**

**Table 4:** Comparison of algorithms on five datasets for Purity.

	ORL	MSRC	HW	Yale	Wikipedia Article
SC (1)	61.63	35.95	77.93	25.08	19.51
SC (2)	50.55	58.00	72.51	41.35	56.46
SC (3)	60.80	54.14	66.15	26.88	-
SC (4)	44.15	63.57	11.13	-	-
SC (5)	-	30.76	66.73	-	-
SC (6)	-	64.19	78.99	-	-
CF	61.03	48.48	65.74	24.68	58.86
AMGL	62.50	78.57	74.88	44.08	41.00
OMSC	49.00	34.29	29.75	16.92	44.16
MVCC	72.25	75.71	85.00	23.09	61.31
MVC-DMF	43.38	39.51	52.88	50.99	51.00
DMFClusts	47.20	50.71	55.58	19.12	28.24
BMVC	59.40	40.10	28.46	29.95	21.31
GFSC	78.38	74.71	82.27	**69.70**	**62.29**
MCLES	81.25	79.52	~	6.67	56.13
MCDCF	75.73	82.76	~	61.39	48.60
RMVCP	**85.00**	**83.81**	**86.90**	61.82	59.88

### 4.4 Weight distribution analysis and convergence speed in the process of RMVCP-2

**Fig 7:**
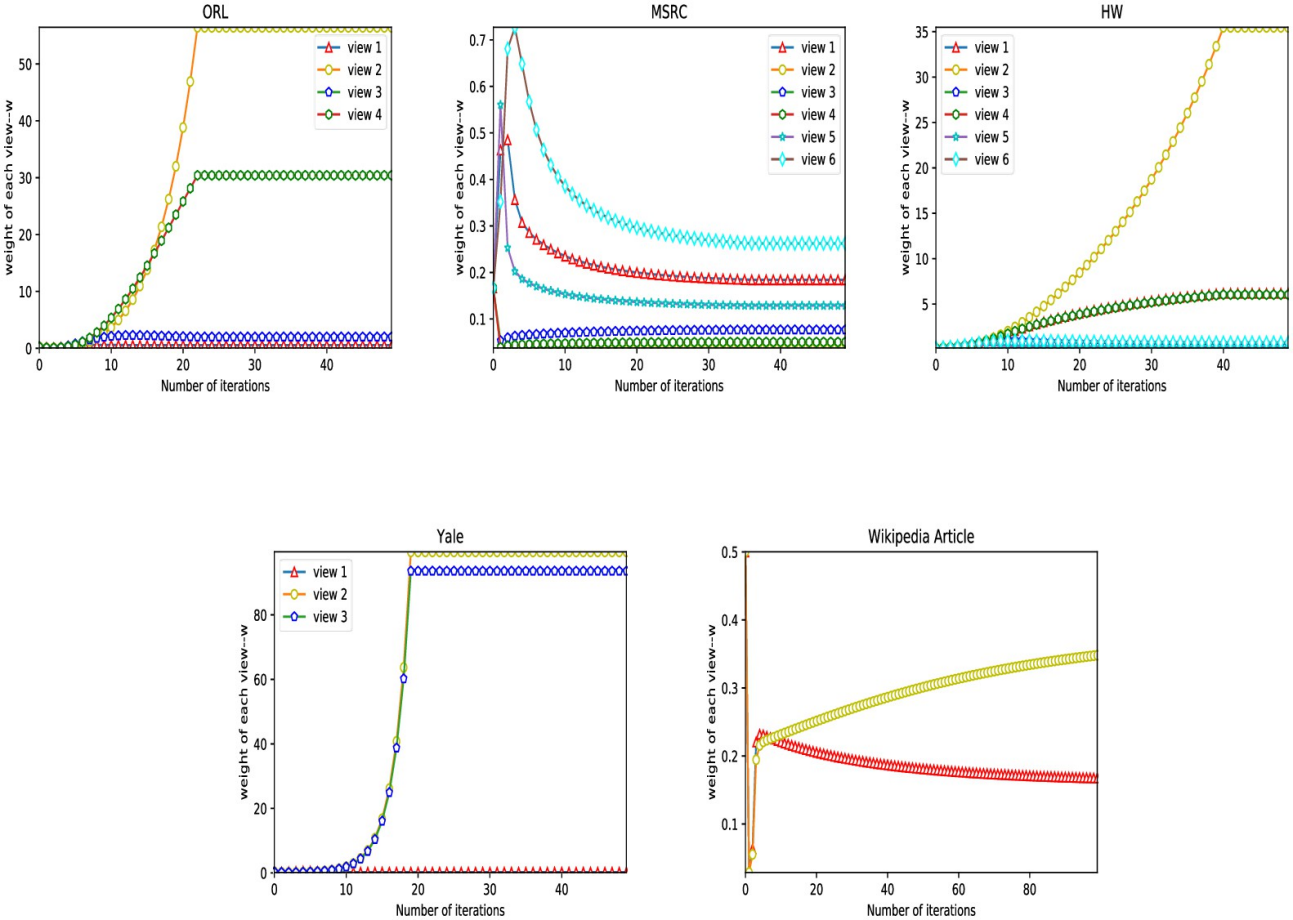
The change of the weight *w* of each view on the five datasets in the RMVCP-1 process.

**Fig 8:**
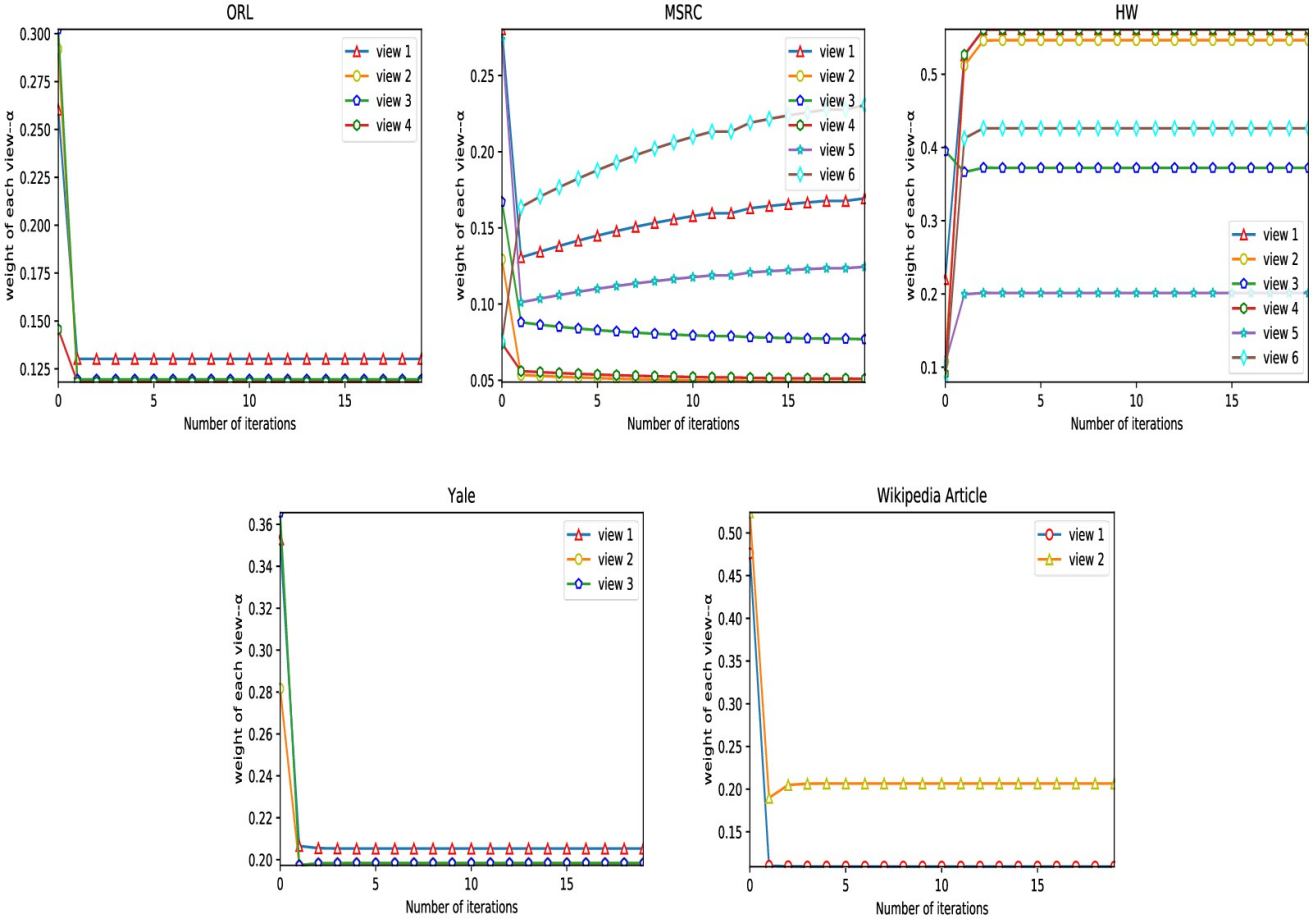
The change of the weight *α*^(*v*)^ of each view on the five datasets in the RMVCP-2 process.

The weight distribution of each view plays a vital role in the clustering performance of multi-view clustering. The RMVCP algorithm will assign the weight of each view twice, which will make up for the defect of unreasonable weight assignment that may be caused by assigning the weight once. Fig 7 shows the change of the weight *w* of each view on the five datasets in the RMVCP-1 process. Fig 8 demonstrates the change of the weight *α*^(*v*)^ of each view on the five datasets in the RMVCP-2 process.

In the RMVCP-1 process, most of the views of the four datasets ORL, MSRC, HW, and Wikipedia Article are assigned weights with higher discrimination. In the RMVCP-2 process, MSRC, HW, and Wikipedia Article also assign a higher discrimination weight to each view, which indicates that on these three data sets, it is not enough to assign weights once to them. A second weight assignment process is needed to achieve a more reasonable weight assignment. In Yale’s two weight assignment processes, the weights of the three views are not much different, which shows that the quality of the three views may be similar. For the ORL data set, the weight distribution in the RMVCP-2 process is not much different, indicating that the data set may be easier to distinguish without the need for two weight distribution processes.

**Fig 9:**
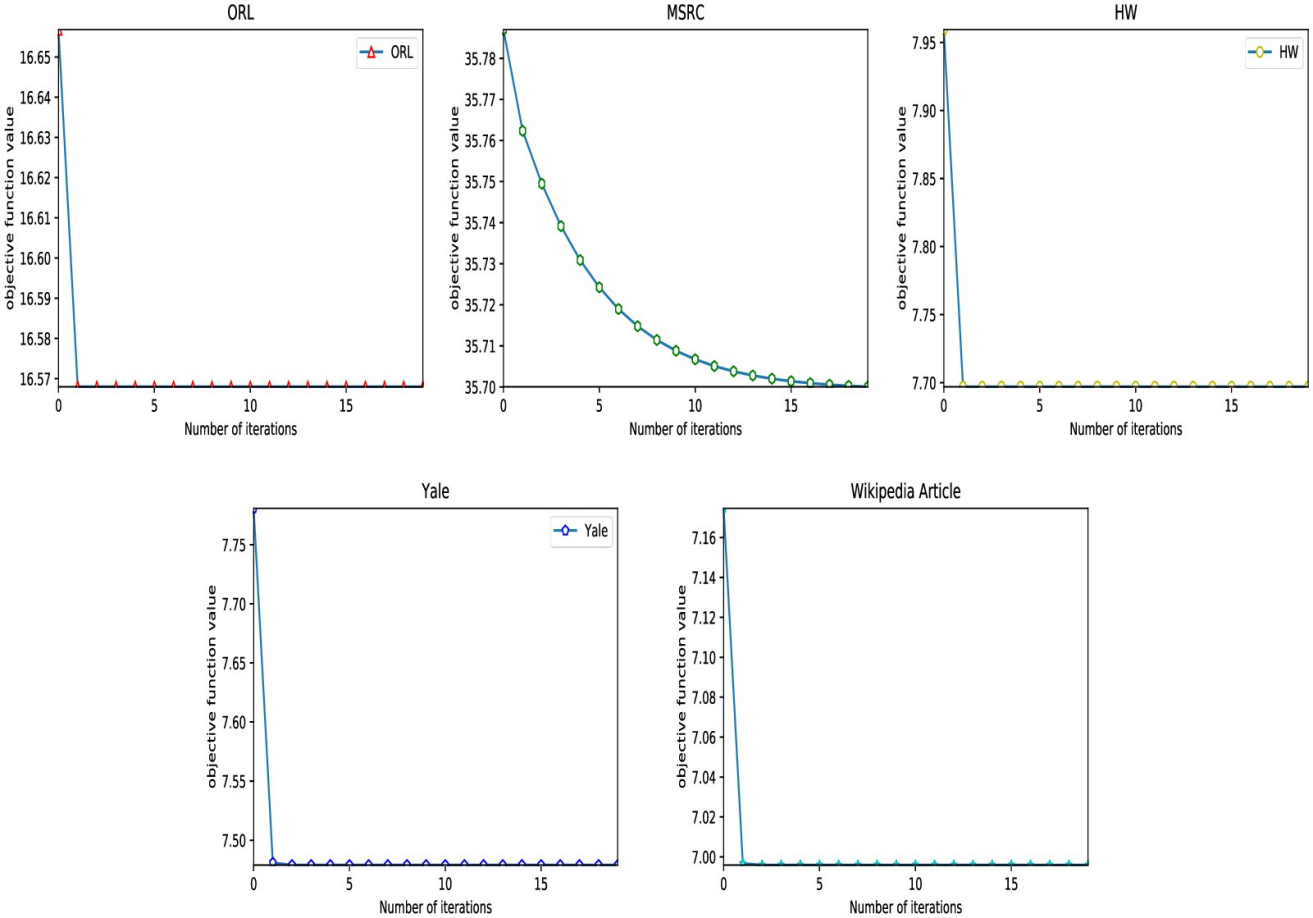
The change of the objective function value of RMVCP-2 on five data sets of ORL, MSRC, HW, Yale, and Wikipedia Article.

Fig 9 shows the change of the objective function value of RMVCP-2 on five data sets of ORL, MSRC, HW, Yale, and Wikipedia Article. Obviously, the convergence speed of the objective function on the five data sets is very fast. The four data sets of ORL, HW, Yale, and Wikipedia Article all converged within five iterations. The MSRC data set has a relatively slow convergence rate, converging around 15 iterations.

### 4.5 Visual analysis of unified matrices U_1_ and U_2_

**Fig 10:**
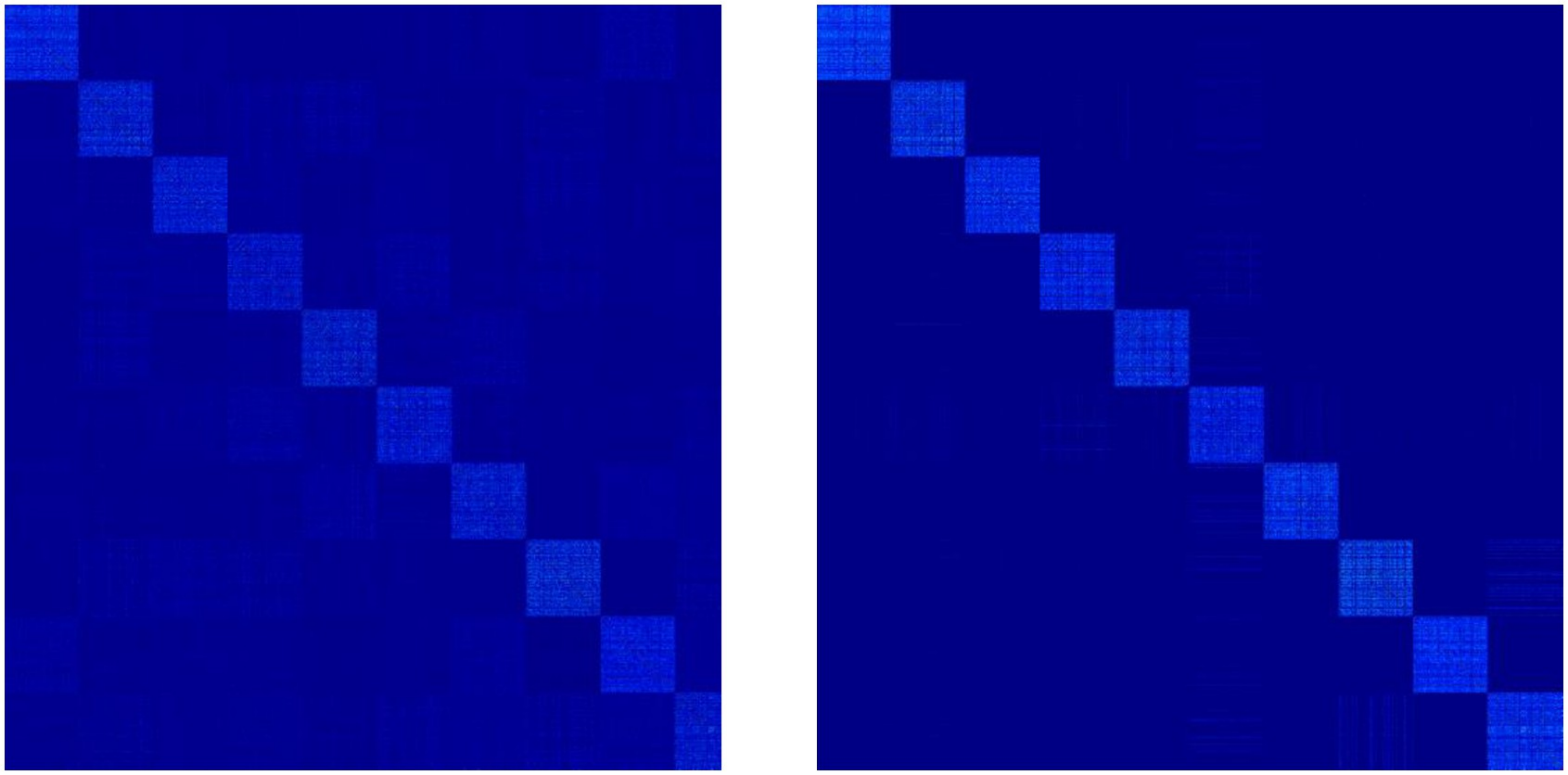
Visual analysis of unified matrices U_1_ and U_2_ on HW.

**Fig 11:**
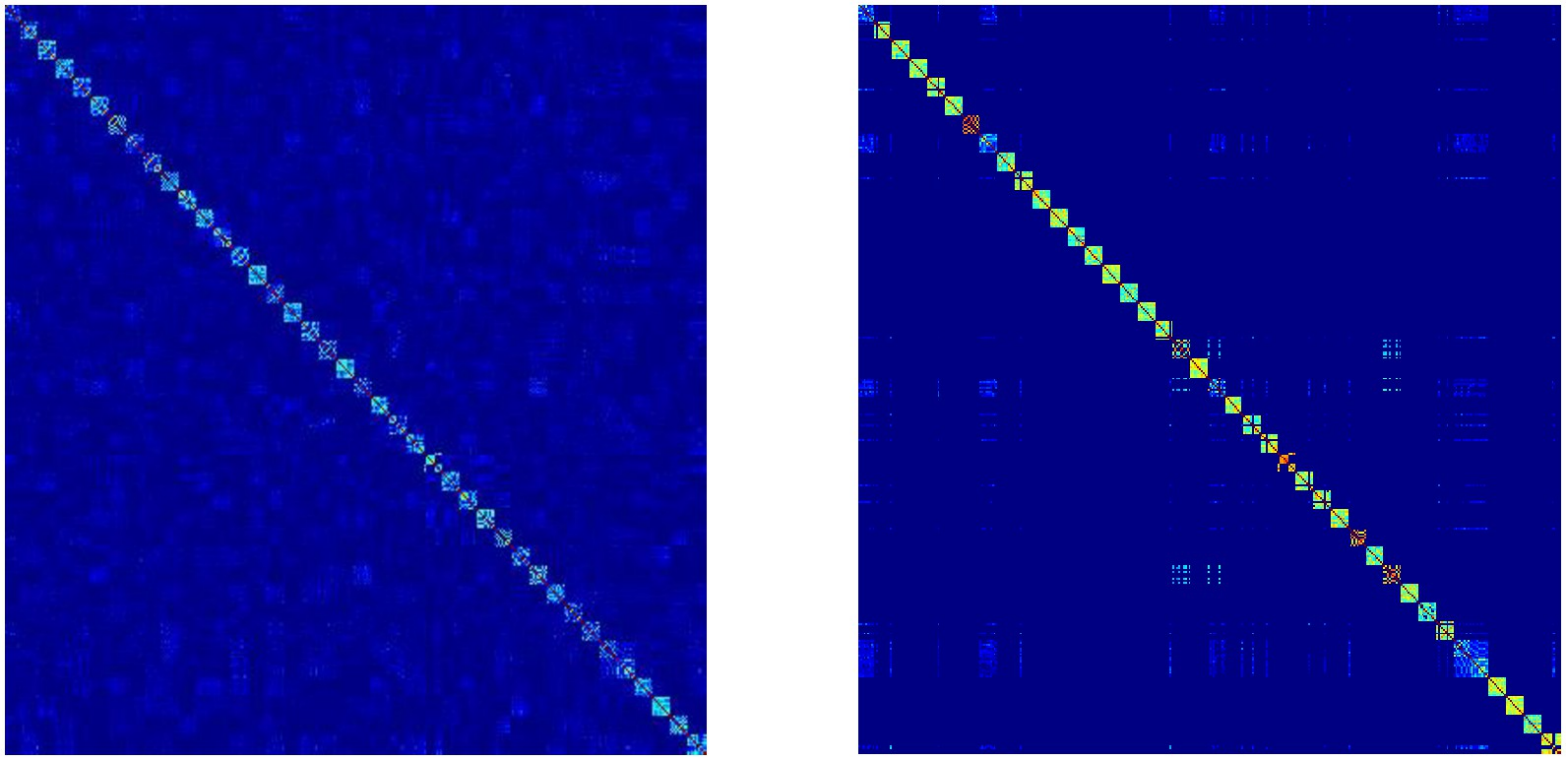
Visual analysis of unified matrices U_1_ and U_2_ on ORL.

To verify the effectiveness of double fusion for weight assignment, we visualize the **U**_1_ produced by the RMVCP-1 process and the **U**_2_ produced by the RMVCP-2 process, respectively. Since both **U**_1_ and **U**_2_ are subjected to the Constrained Laplacian Rank operation, the better the clustering performance, the clearer the block structure of **U**_1_ and **U**_2_. Figs 10 and 11 shows the block structure of **U**_1_ and **U**_2_ on HW and ORL. It is obvious from Figs 10 and 11 that **U**_2_ has a clearer block structure and less noisy data than **U**_1_. This shows that RMVCP-2 is a necessary and effective step to improve clustering performance due to its better weight assignment of views and reduction of noisy data.

### 4.6 Parameter analysis

**Fig 12:**
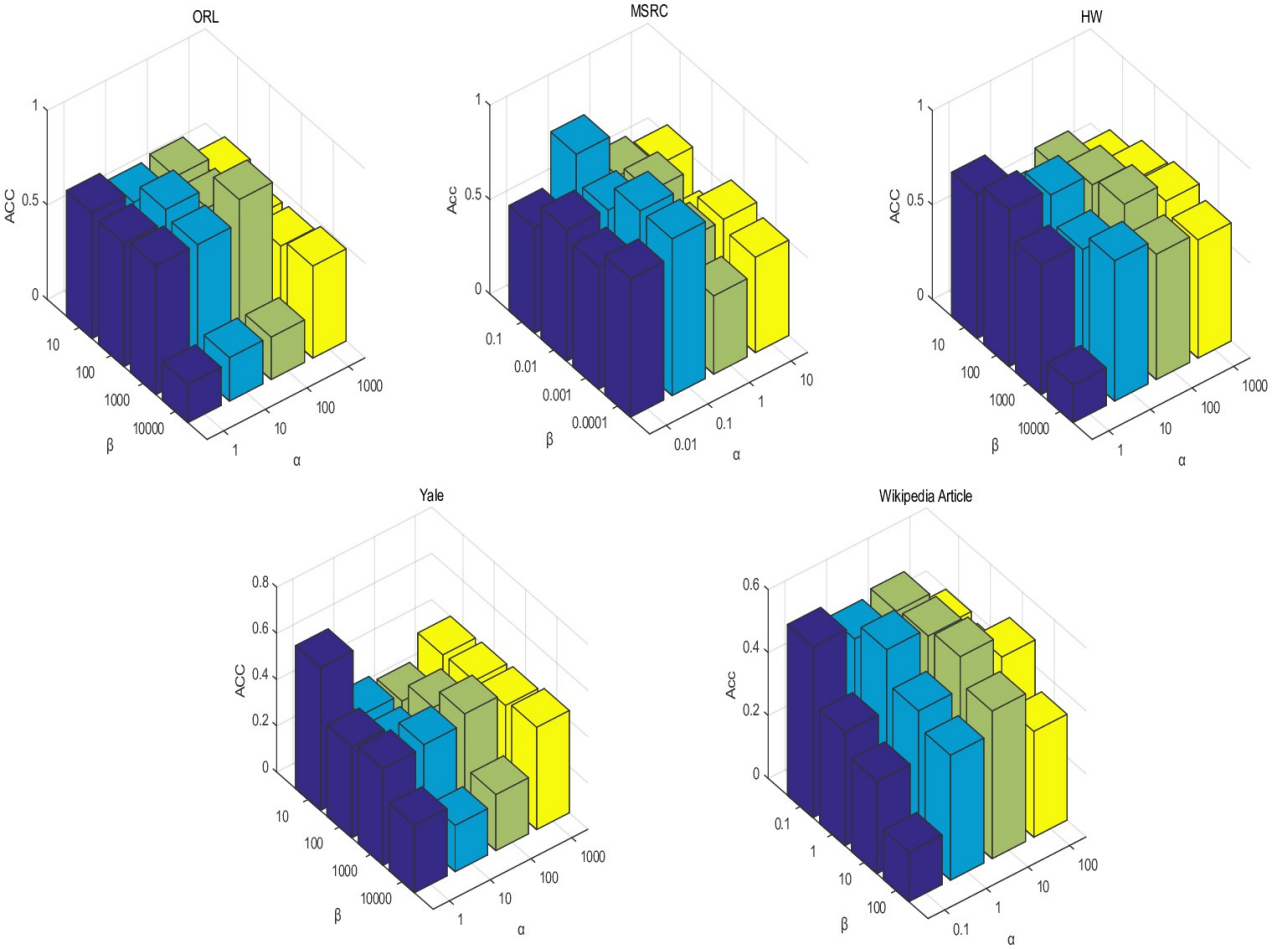
The influence of the changes of hyperparameters *α*, *β*, and *γ* on the results of Acc on the five datasets.

There are three hyperparameters in the RMVCP algorithm, namely *α*, *β*, and *γ*, all of which need to be set in advance before the experiment. The value of *γ* will not change the clustering results in the actual experiment. We set *γ* to 0.01 in the experiment. Fig 12 shows the influence of the changes of hyperparameters *α*, *β*, and *γ* on the results of Acc on the five datasets. After doing a lot of experiments, we found that the best hyperparameters *α*, *β*, and *γ* on ORL are set to 100, 1000, and 0.01. On MSRC, the parameters are set to 0.1, 0.1, 0.1. The parameter setting on HW is 1,100,0.01. The parameter is set to 1, 10, 0.01 on Yale. The parameters on Wikipedia Article are set to 1, 1, 0.01.

## 5. Discussion

Extensive experiments have verified that the clustering performance of RMVCP algorithm is better than that of other state-of-the-art algorithms, indicating the effectiveness of twice weight allocation for each view and combination with tissue-like P system. The quality of each view is irregularity, and RMVCP performs two weight assignment operations on each view. The results and effects are revealed in the experiment to verify its effectiveness. In addition to the better accuracy of the RMVCP algorithm, RMVCP can also handle a slightly larger dataset rather than MCLES and MCDCF. However, three parameters need to be set in advance in RMVCP algorithm. It can be seen from parameter sensitivity experiment that RMVCP algorithm is sensitive to *α* and *β* on some datasets, which is the deficiency of RMVCP algorithm. Therefore, we will focus on the parameter problem of the algorithm in the future, and strive to reduce the number of parameters and weaken the influence of different parameter values on clustering performance.

## 6. Conclusion and future research

In this paper, in order to solve the problem of view weight distribution and noise reduction in multi-view clustering, Reweighted multi-view clustering with tissue-like P system (RMVCP) are proposed. Inspired by multi-view subspace clustering and graph-based multi-view clustering, RMVCP performs a two-step operation. In the first step (RMVCP-1), the self-representation method is used to construct the similarity matrix of each view, and then the fusion operation is performed. In the second step (RMVCP-2), the updated similarity matrix of each view generated in the process of RMVCP-1 is used as input for the second fusion operation. Correspondingly, the weight of each view has been allocated more reasonably. At the same time, we combine the RMVCP algorithm with the tissue-like P system, and use the computational parallelism of the tissue-like P system to improve the computational efficiency of the RMVCP algorithm. In the future, we can use the idea of secondary fusion in some other state-of-the-art multi-view clustering algorithms, and at the same time, we can combine multiple models in membrane computing with clustering algorithms.

## Supporting information

S1 File 5 datasets are used in the experiment in this paper. (ZIP)
